# Resectability, conversion, metastasectomy and outcome according to *RAS* and *BRAF* status for metastatic colorectal cancer in the prospective RAXO study

**DOI:** 10.1038/s41416-022-01858-8

**Published:** 2022-05-24

**Authors:** Aki Uutela, Emerik Osterlund, Päivi Halonen, Raija Kallio, Annika Ålgars, Tapio Salminen, Annamarja Lamminmäki, Leena-Maija Soveri, Raija Ristamäki, Kaisa Lehtomäki, Hanna Stedt, Eetu Heervä, Timo Muhonen, Juha Kononen, Arno Nordin, Ali Ovissi, Soili Kytölä, Mauri Keinänen, Jari Sundström, Lasse Nieminen, Markus J. Mäkinen, Teijo Kuopio, Ari Ristimäki, Helena Isoniemi, Pia Osterlund

**Affiliations:** 1 Transplantation and Liver Surgery, Abdominal Center, Helsinki University Hospital and University of Helsinki, Helsinki, Finland; 2 Department of Immunology, Genetics and Pathology, Uppsala University, Uppsala, Sweden; 3 Department of Oncology, Helsinki University Hospital Comprehensive Cancer Center and University of Helsinki, Helsinki, Finland; 4 Department of Oncology, Oulu University Hospital, Oulu, Finland; 5 Department of Oncology, Turku University Hospital and University of Turku, Turku, Finland; 6 Department of Oncology, Tampere University Hospital and University of Tampere, Tampere, Finland; 7 Department of Oncology, Kuopio University Hospital and University of Eastern Finland, Kuopio, Finland; 8 Joint Municipal Authority for Health Care and Social Services in Keski-Uusimaa, Home Care Geriatric Clinic and Palliative Care, Hyvinkää, Finland; 9 Department of Oncology, South Carelia Central Hospital, Lappeenranta, Finland; 10 Department of Oncology, Central Finland Hospital Nova, Jyväskylä, Finland; 11 Docrates Cancer Center, Helsinki, Finland; 12 Department of Radiology, HUS Medical Imaging Centre, Helsinki University Hospital and University of Helsinki, Helsinki, Finland; 13 Department of Genetics, HUSLAB, HUS Diagnostic Center, Helsinki University Hospital and University of Helsinki, Helsinki, Finland; 14 Department of Genetics, FIMLAB laboratories, Tampere University Hospital, Tampere, Finland; 15 Department of Pathology, Turku University Hospital and University of Turku, Turku, Finland; 16 Department of Pathology, Tampere University Hospital and University of Tampere, Tampere, Finland; 17 Department of Pathology, Oulu University Hospital and University of Oulu, Oulu, Finland; 18 Department of Pathology, Central Finland Hospital Nova and Department of Biological and Environmental Science, University of Jyväskylä, Jyväskylä, Finland; 19 Department of Pathology, HUSLAB, HUS Diagnostic Centre and Applied Tumour Genomics, Research Programs Unit, Helsinki University Hospital and University of Helsinki, Helsinki, Finland; 20 Department of Oncology/ Oncology-Pathology, Karolinska University Hospital Comprehensive Cancer Center, and Karolinska Institutet, Stockholm, Sweden

**Keywords:** Metastasis, Colorectal cancer, Surgical oncology, Prognostic markers

## Abstract

**Background:**

Outcomes after metastasectomy for metastatic colorectal cancer (mCRC) vary with *RAS* and *BRAF* mutational status, but their effects on resectability and conversion rates have not been extensively studied.

**Methods:**

This substudy of the prospective RAXO trial included 906 patients recruited between 2011 and 2018. We evaluated repeated centralised resectability assessment, conversion/resection rates and overall survival (OS), according to *RAS* and *BRAF* status.

**Results:**

Patients included 289 with *RAS* and *BRAF* wild-type (*RAS* and *BRAF*wt), 529 with *RAS* mutated (*RAS*mt) and 88 with *BRAF* mutated (*BRAF*mt) mCRC. Metastatic prevalence varied between the *RAS* and *BRAF*wt/*RAS*mt/*BRAF*mt groups, for liver (78%/74%/61%), lung (24%/35%/28%) and peritoneal (15%/15%/32%) metastases, respectively. Upfront resectability (32%/29%/15%), conversion (16%/13%/7%) and resection/local ablative therapy (LAT) rates (45%/37%/17%) varied for *RAS*a and *BRAF*wt/*RAS*mt/*BRAF*mt, respectively. Median OS for patients treated with resection/LAT (*n* = 342) was 83/69/30 months, with 5-year OS-rates of 67%/60%/24%, while systemic therapy-only patients (*n* = 564) had OS of 29/21/15 months with 5-year OS-rates of 11%/6%/2% in *RAS* and *BRAF*wt/*RAS*mt/*BRAF*mt, respectively. Resection/LAT was associated with improved OS in all subgroups.

**Conclusions:**

There were significant differences in resectability, conversion and resection/LAT rates according to *RAS* and *BRAF* status. OS was also significantly longer for *RAS* and *BRAF*wt versus either mutant. Patients only receiving systemic therapy had poorer long-term survival, with variation according to molecular status.

**Clinical trial registration:**

NCT01531621/EudraCT2011-003158-24

## Background

The majority of colorectal cancer (CRC) tumours develop through chromosomal instability and mutations in tumour suppressor genes and oncogenes [1]. *RAS* oncogene mutations are found in about 50% of CRC tumours with *KRAS* being the dominant and *NRAS* less frequent, while *BRAF* mutations are reported in 5–10% of tumours, with up to 21% reported in cohorts of patients with unresectable metastatic CRC (mCRC) [2, 3].

The *RAS* and *BRAF* genes encode proteins that mediate intracellular signalling pathways downstream of the epithelial growth factor receptor (EGFR) [4]. These mutations cause resistance to EGFR therapeutic antibodies and *RAS* and *BRAF* testing is now recommended for all patients with mCRC [2, 5–7]. *BRAF*-V600E is the dominant *BRAF* mutation in CRC, found in more than 90% of patients with a *BRAF* mutated type (*BRAF*mt) gene [8]. *BRAF*mt is associated with higher tumour grade, right-sided primary tumours, female gender, older age, deficient mismatch repair (dMMR) status and higher prevalence of peritoneal and lymph node metastases [4]. *RAS* mutated type (*RAS*mt) genes have been linked to a higher prevalence of lung metastases [4].

Both *RAS* and *BRAF* mutations have been associated with worse survival after resection of CRC liver metastases [9, 10]. For *BRAF* mutations even the rationale for resecting patients with mCRC has been questioned [11], although some encouraging reports of long-term survival have also been published [12, 13]. Outcomes after metastasectomy vary with *RAS* and *BRAF* status, but the significance of these mutations in the setting of multiorgan metastatic disease and resection with curative intent is still unclear.

The aim of this study was to evaluate how *RAS* and *BRAF* mutational status affects resectability, conversion and resection rates, differences in metastatic profile and overall survival (OS) after resection and/or local ablative therapy (LAT) and systemic therapy in patients with mCRC.

## Methods

### Study design

The prospective, investigator-initiated, nationwide Finnish RAXO-study (NCT01531621, EudraCT 2011-003158-24) included 1086 patients with metastatic colorectal cancer recruited from 2012 to 2018 [14, 15]. The oncology departments of all 5 university hospitals and all 16 regional hospitals in Finland participated in the study. Inclusion criteria were patients eligible for first-line systemic therapy, age over 18 years and histologically confirmed colorectal adenocarcinoma with distant metastases or locally advanced primary tumours not curatively treatable. The main protocol has been published in detail [14]. A part of this substudy was presented as a poster and oral presentation at the American Society of Clinical Oncology’s (ASCO) Annual Meeting, 4–8 June 2021 [16].

### Patients

Of the whole RAXO-study population, we excluded patients not accurately defined as *RAS* and *BRAF* wild-type (*RAS* and *BRAF*wt) because of incomplete mutational analysis, i.e. no *KRAS*, *NRAS* of *BRAF* mutation found but all mutations not tested (*n* = 155), those who received only best supportive care (*n* = 17) or who had an atypical *BRAF* (non-V600E) mutation (*n* = 8). The remaining 906 patients formed the cohort for this substudy that was used to evaluate the secondary aim of prognostic and predictive biomarkers. Computed tomography (CT) for detecting the extent of disease was often done after the diagnostic colonoscopy, and thus even truly synchronous metastases were often detected only shortly after the primary tumour. Because of this, metastases were considered to be synchronous, if they were detected before or within two months of diagnosis of the primary tumour. The data cut-off date for follow-up was 27 March 2020. At that time 609 of the patients (67%) were deceased, mostly due to progressive mCRC.

### Molecular pathology

*KRAS*, *NRAS* and *BRAF-*V600E mutations were analysed with reverse transcriptase-polymerase chain reaction (PCR) in 42%, Next-generation sequencing (NGS) in 45%, Idylla panels in 12% and Sanger sequencing and pyrosequencing in 1%, from either histological biopsy or resection specimen from the primary tumour or liver metastasis. The *RAS* and *BRAF*wt were tested for *KRAS* and *NRAS* exons 2–4 and *BRAF*-V600E. Of the *RAS*mt patients 294 were not analysed but assumed *BRAF*wt as the coexistence of *RAS* and *BRAF* mutations was considered very rare [4]. Immunohistochemistry with primary antibodies for MLH1, MSH2, MSH6 and PMS2 proteins was used to identify deficient mismatch repair status, and PCR was used when the results of immunohistochemistry were indeterminate.

### Systemic therapy

Standard local treatment protocols based on ESMO [5] and NCCN [6, 7] guidelines were used for systemic therapy, which was given until disease progression, unacceptable toxicity or resection/LAT was achieved. In the perioperative setting mainly oxaliplatin and fluoropyrimidine-based treatment regimens were used [17]. For conversion chemotherapy the most intensive regimen was used, preferably a doublet or triplet chemotherapy combined with a targeted agent (bevacizumab, cetuximab or panitumumab) based on *RAS* and *BRAF* status [5].

### Resectability assessment

The first local resectability assessment was done at the local hospital before starting first-line treatment, often before recruitment to the RAXO-study. After study inclusion, baseline demographics were provided online and the multidisciplinary team (MDT) at Helsinki University Hospital tertiary centre evaluated the technical resectability of the liver, lung and other metastases based on imaging. The imaging examinations included whole body (chest, abdomen and pelvis) CT supplemented by magnetic resonance imaging and 18F-fluoro-deoxyglucose positron emission tomography (PET) as needed. The MDT consisted of experts in liver surgery and abdominal radiology with medical oncologists, radiation oncologists, colorectal and cytoreductive surgeons, thoracic surgeons, gynaecologists, thoracic radiologists and PET specialists, as required. The MDT assessment was performed on baseline radiology (when the metastatic disease was noted) and repeated twice, if needed, after 2–3 and 4–6 months of systemic therapy and provided electronically to the treating physicians. The treatment decisions were made by local or central MDTs and resections were mostly carried out at specialised centres at the six largest hospitals.

### Statistical analysis

For differences in demographics and other nominal factors, Bonferroni correction for Chi-square analyses per variable was applied. For variables with significant differences logistic regression was used to calculate odds ratios (OR) with 95% confidence intervals (95% CI). The Kaplan–Meier method was used to estimate OS, which was calculated from the diagnosis of metastatic disease to the date of death or censored at the last follow-up. Conditional 12-month Landmark analysis of OS was used to control a potential guarantee-time bias as reported previously [15]. Hazard ratios (HR) and corresponding 95% CI for survival were calculated using Cox proportional hazard regression. Univariate analyses were first performed and variables with significant HR were then entered into the multivariable analysis. The study had 609 OS events which allowed for several covariates in multivariable analysis. The median follow-up time was calculated with the reverse Kaplan–Meier method. All analyses were carried out using SPSS Statistics, Version 25.0, Armonk, NY.

## Results

The molecular substudy included 906 patients, of which 289 were *RAS* and *BRAF*wt (32%), 529 were *RAS*mt (58%) and 88 were *BRAF*-V600Emt (10%). *KRAS*mt (*n* = 491, 54%) and *NRAS*mt (*n* = 38, 4%) patients were analysed as one group (*RAS*mt). Mismatch repair status was examined in 294 patients, 12 of whom had dMMR (4%), including 2 *RAS* and *BRAF*wt, 5 *RAS*mt and 5 *BRAF*mt. The median follow-up time was 55 months, with a minimum follow-up of 18 months.

The median age of the treatable patients was 66 years. Demographics (Table 1) showed that *BRAF*mt tumours were more common among women (OR 3.3) and in ECOG performance status 2–3 patients (OR 1.5) with *RAS* and *BRAF*wt as reference (ref) in all analyses. Primaries with *BRAF*mt were predominantly right-sided (OR 11.8), whereas *RAS* and *BRAF*wt tumours were mostly left-sided (either colon or rectum), and *RAS*mt patients’ tumours were in between (OR 2.3). Tumours with *BRAF*mt had more often signet cell or mucinous histology (OR 4.2), and this was also more likely for *RAS*mt tumours (OR 1.7) than for *RAS* and *BRAF*wt. *RAS*mt patients received adjuvant therapy after resection of the primary tumour less often than *RAS* and *BRAF*wt (OR 0.7). No differences were noted in Charlson comorbidity index, low body mass index (BMI), surgery of the primary tumour or synchronous presentation.

**Table 1:** Patient demographics.

	Total		*RAS* and *BRAF*wt		*RAS*mt		*BRAF*mt	
	906	100%	289	100%	529	100%	88	100%
Age								
Median years (range)	66.1	(24–88)	65.8	(24–88)	66.1	(25–88)	66.9	(33–83)
≤70	607	67%	201	70%	347	66%	59	67%
>70	299	33%	88	30%	182	34%	29	33%
Sex								
Male	549	61%	195	68%^b^	320	61%^b^	34	39%^b^
Female	357	39%	94	33%^b^	209	40%^b^	54	61%^b^
ECOG								
PS 0	260	29%	84	29%	157	30%	19	22%
PS 1	503	56%	160	55%	296	56%	47	53%
PS 2–3	143	16%	45	16%^c^	76	14%^c^	22	25%^c^
Charlson comorbidity index								
0	700	77%	226	78%	409	77%	65	74%
1–2	199	22%	60	21%	116	22%	23	26%
3–5	7	1%	3	1%	4	1%	0	0%
Body mass index								
<20	64	7%	19	7%	40	8%	5	6%
20–30	674	74%	219	76%	392	74%	63	72%
≥30	168	19%	51	18%	97	18%	20	23%
Primary tumour location								
Right colon	261	29%	45	16%^d^	157	30%^d^	59	67%^d^
Left colon	330	36%	134	46%^d^	181	34%^d^	15	17%^d^
Rectum	310	34%	108	37%^d^	190	36%^d^	12	14%^d^
Multiple	5	1%	2	1%	1	0%	2	2%
Signet ring or mucinous carcinoma								
No	269	93%	469	89%	805	89%	67	76%
Yes	20	7%^e^	60	11%^e^	101	11%^e^	21	24%^e^
Primary tumour resection								
Upfront	604	67%	195	68%	350	66%	59	67%
During	96	11%	39	14%	51	10%	6	7%
No	206	23%	55	19%	128	24%	23	26%
Presentation of metastases								
Synchronous^a^	608	67%	180	62%	366	69%	62	71%
Metachronous	298	33%	109	38%	163	31%	26	30%
Adjuvant chemotherapy for primary tumour								
No adjuvant	690	76%^f^	205	71%^f^	417	79%^f^	68	77%^f^
Fluoropyrimidine	91	10%	35	12%	51	10%	5	6%
Oxaliplatin based	125	14%	49	17%	61	12%	15	17%
Radiotherapy for rectum								
No	192	62%	70	65%	115	61%	7	58%
Preop 5 × 5 Gy	46	15%	14	13%	32	17%	0	0%
Chemoradiation	54	17%	17	16%	33	17%	4	33%
Palliative	18	6%	7	6%	10	5%	1	8%
Metastatic sites								
Single	483	53%	152	53%	285	54%	46	52%
Multiple	423	47%	137	47%	244	46%	42	48%
Location of metastases at baseline								
Liver	675	75%	224	78%^g^	394	74%^g^	57	65%^g^
Lung	278	31%	68	24%^h^	185	35%^h^	25	28%^h^
Lymph nodes	235	26%	83	29%	123	23%	29	33%
Peritoneal	151	17%	43	15%^i^	80	15%^i^	28	32%^i^
Local relapse	55	6%	20	7%	26	5%	9	10%
Other	121	13%	50	17%	61	11%	10	11%

OR (95% CI), respectively, for *RAS* and *BRAF*wt /*RAS*mt/*BRAF*mt.

^a^Within 2 months from the diagnosis of primary tumour.

^b^For female sex ref/1.4(1.0–1.8)/3.3(2.0–5.4).

^c^For ECOG PS 2–3 vs 0–1 ref/0.96(0.77–1.12)/1.5(1.0–2.1).

^d^For more right-sided tumours than left-sided or rectal (multifocal excluded) ref/2.3(1.6–3.3)/11.8(6.7–20.5).

^e^For signet ring or mucinous carcinoma ref/1.7(1.0–2.9)/4.2(2.2–8.2).

^f^For adjuvant therapy after resection of primary tumour ref/0.7(0.5–0.9)/0.7(0.4–1.3).

^g^For liver metastases more common ref/0.8(0.6–1.2)/0.5(0.3–0.6).

^h^For lung metastases more common ref/1.7(1.3–2.4)/1.3(0.8–2.2).

^i^For peritoneal metastases more common ref/1.0(0.7–1.5)/2.7(1.6–4.6).

**Fig. 1:**
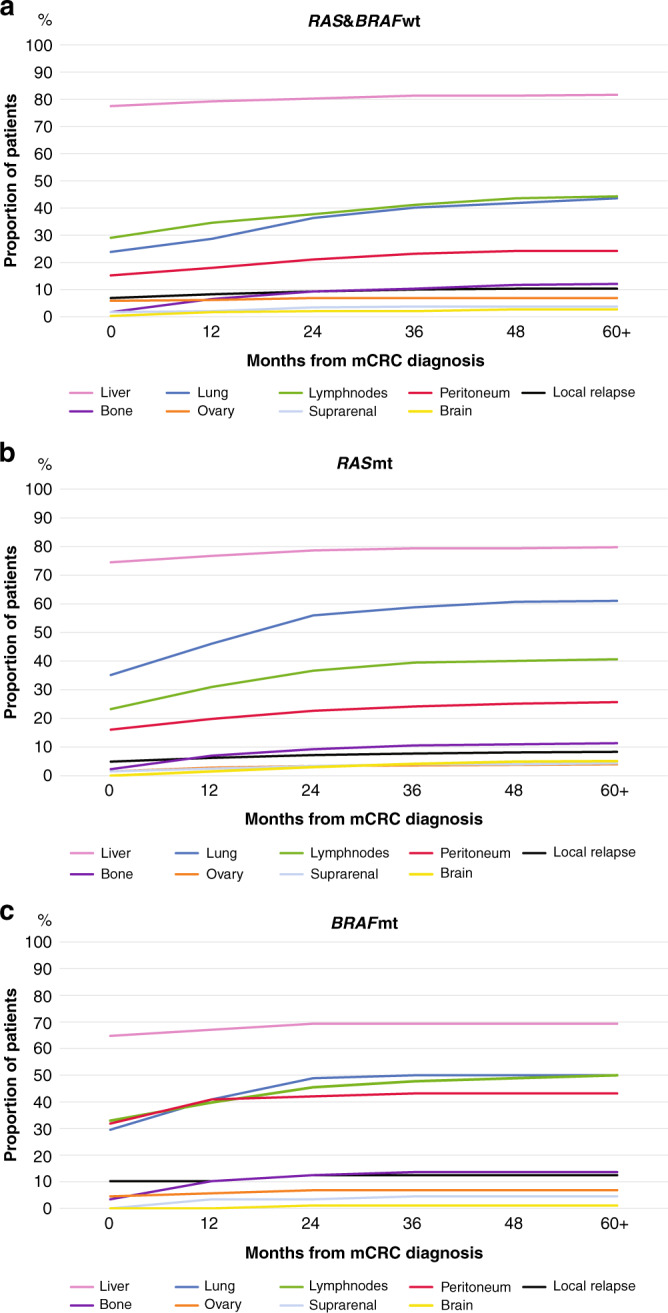
Metastatic sites at baseline and during disease trajectory (months). **a** *RAS* and *BRAF* wild-type. **b** *RAS* mutated type. **c** *BRAF* mutated type.

The metastatic profiles at baseline, when mCRC was diagnosed, were different according to mutational status (Table 1, Fig. 1). Liver metastases were less common for *BRAF*mt (OR 0.5) than *RAS* and *BRAF*wt patients. *RAS*mt patients were more likely to have lung metastases than *RAS* and *BRAF*wt (OR 1.7). Peritoneal metastases were more common in *BRAF*mt (OR 2.7) than for *RAS* and *BRAF*wt (ref) or *RAS*mt (OR 1.0) patients. No differences were observed for distant lymph node metastases, for other metastatic sites or for the number of metastatic sites.

**Fig. 2:**
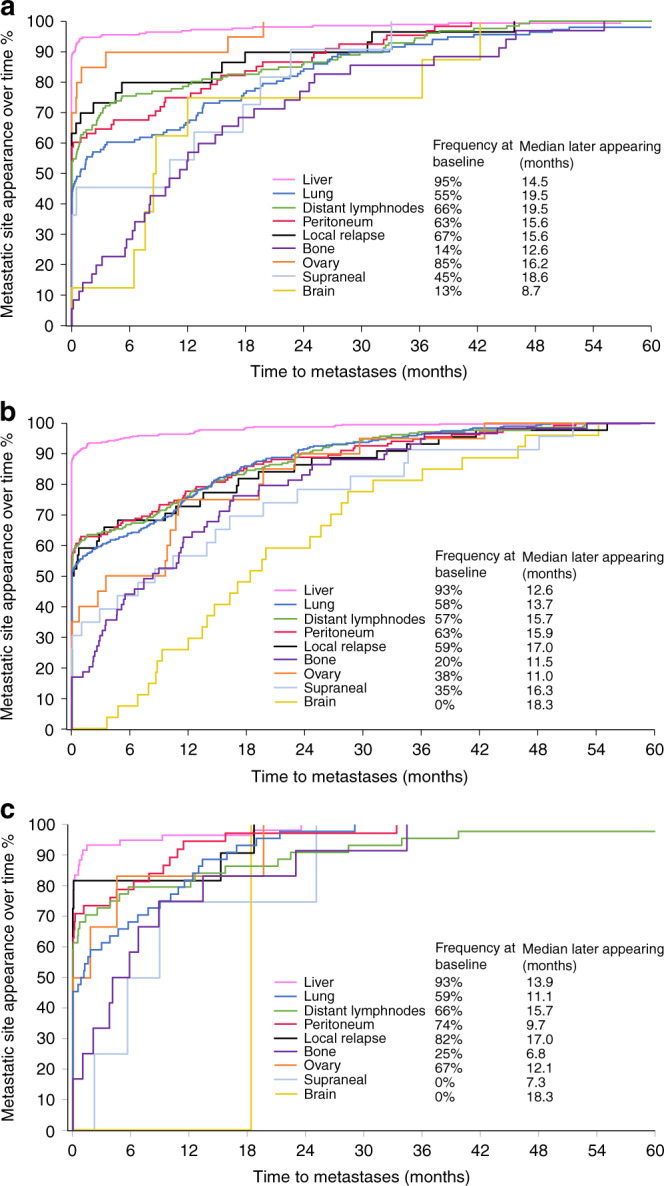
Appearance of the metastatic sites over time for patients who were diagnosed with metastases in specified organs during trajectory. **a** *RAS* and *BRAF* wild-type. **b** *RAS* mutated type. **c** *BRAF* mutated type.

The liver was the most common metastatic site at the time of diagnosis of metastatic disease as well as throughout the disease trajectory (Fig. 1). Liver metastases during disease trajectory were more likely for *RAS* and *BRAF*wt (82%, ref) and *RAS*mt (80%, OR 1.1 [95% CI 0.8–1.6]) compared with *BRAF*mt patients (69%, 0.6 [0.3–0.9]). Lung metastases during disease trajectory were more common for *RAS*mt (61%, 2.0 [1.5–2.7]) than for *RAS* and *BRAF*wt (44%, ref) with *BRAF*mt patients in between (50%, 1.3[0.8–2.1]). Peritoneal metastases during trajectory were more common among *BRAF*mt (43%, 2.4 [1.4–3.9]) and less common among *RAS*mt (26%, 1.1 [0.7–1.5]), and *RAS* and *BRAF*wt patients (24%, ref). There were no significant differences in the prevalence of lymph node, bone, ovarian, suprarenal or brain metastases, and local relapse between the molecular subtypes. Bone, brain and suprarenal metastases were more likely to appear later during the disease (Fig. 2).

**Fig. 3:**
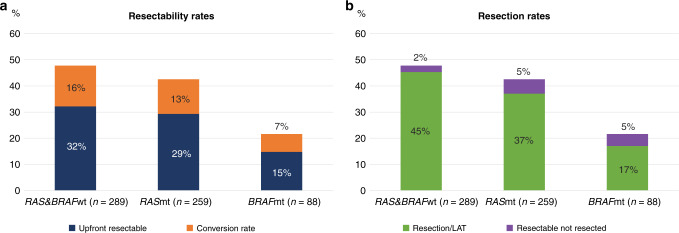
Resectability and resections. **a** Central Resectability and conversion rates (% of entire cohort) according to RAS and BRAF mutational status. **b** Corresponding resection rates (% of entire cohort).

The likelihood of upfront resectability of all metastatic sites in central assessment (Fig. 3) was lower for *BRAF*mt (15%, OR 0.3 [0.2–0.6]) than for *RAS*mt (29%, OR 0.8 [0.6–1.1]) or *RAS* and *BRAFwt* (32%, ref). For borderline resectable, conversion to resectable with systemic therapy was higher for *RAS* and *BRAFwt* (23%, ref) and *RAS*mt (19%, OR 0.8 [0.5–1.2]) than for *BRAF*mt (8%, OR 0.3 [0.1–0.7]). The overall resectability rates (including conversion) were higher for *RAS* and *BRAF*wt (48%, ref) and *RAS*mt (43%, OR 0.8 [0.6–1.1]) than for *BRAF*mt (22%, OR 0.3 [0.2–0.5])

**Fig. 4:**
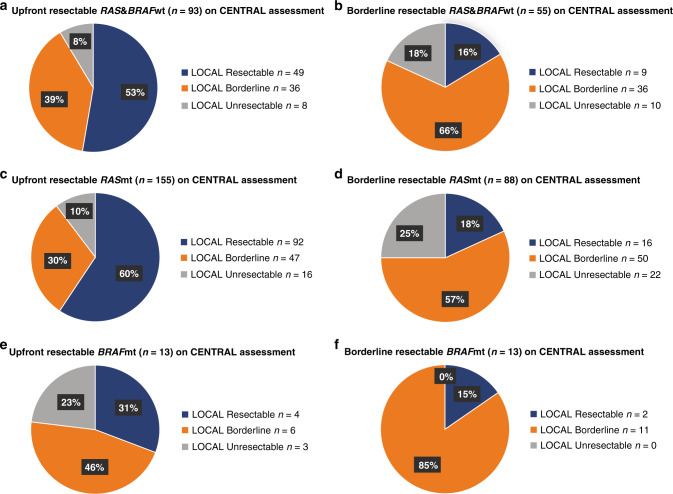
Upfront resectable (left panels) and borderline resectable (right panels) in the central tertiary centre multidisciplinary team resectability assessment compared with resectability assessment in local hospitals done before systemic therapy and recruitment to the RAXO trial. **a** and **b** *RAS* and *BRAF* wild type patients. **c** and **d** *RAS* mutated type patients. **e** and **f** *BRAF* mutated type patients.

There were differences in upfront resectability assessment between centralised tertiary MDT and local evaluation (Fig. 4). The discrepancy was highest in patients centrally classified as upfront resectable. The underestimation of upfront resectability was 47% for *RAS* and *BRAF*wt, 40% for *RAS*mt and 69% for *BRAF*mt. When the central assessment was borderline resectable, the local assessment was concordant in 57–85% of cases, but even then, up to 25% of patients were locally considered completely unresectable.

In total, 342 (38% of 906) patients were resected and/or treated with LAT. Resection and/or LAT rates were highest for *RAS* and *BRAF*wt (45%, ref), slightly lower for *RAS*mt (37%, OR 0.7 [0.5–1.0]) and lowest for *BRAF*mt (17%, OR 0.2 [0.1–0.5]).

**Fig. 5:**
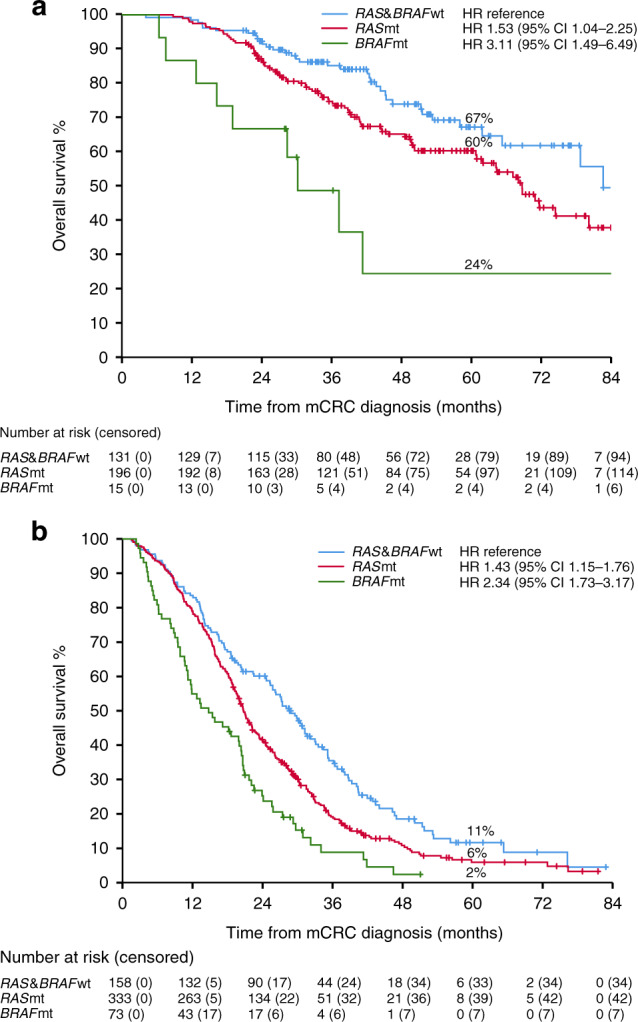
Overall survival from diagnosis of metastatic disease. **a** Patients who were resected and/or treated with local ablative therapy. **b** Patients who received systemic therapy only.

Patients with *RAS* and *BRAF*wt tumours had the longest mOS after the diagnosis of metastatic disease of 83 months with a 5-year OS-rate of 67% in resected and/or LAT treated and *RAS*mt patients had a mOS of 69 months, with a 5-year OS-rate of 60% (HR 1.53 [95% CI 1.04–1.76], Fig. 5). *BRAF*mt patients had a shorter mOS of 30 months with a 5-year OS-rate of 24% with resection and/or LAT (HR 3.11 [95% CI 1.49–6.49] vs *RAS* and *BRAF*wt).

At mCRC diagnosis 46–48% of patients presented with multiple metastatic sites (2–6 sites) with no differences between mutational groups. The resected and/or LAT patients had a better OS than those not resected (Supplementary Fig. 1). Only two *BRAF*mt patients with multiple metastatic sites were resected.

OS in “systemic therapy only” patients (*n* = 564, 62%) was longest for *RAS* and *BRAF*wt (29 months), intermediate for *RAS*mt (21 months) and shortest for *BRAF*mt (15 months). The 5-year OS-rates were 11%, 6% and 2%, respectively (Fig. 5). Patients who only received systemic therapy had worse survival than resected and/or LAT patients in all the mutational groups (Supplementary Fig. 2). Similar results were also seen in a 12-month conditional Landmark analysis of OS for *RAS* and *BRAF*wt and *RAS*mt (Supplementary Fig. 3).

Prognostic baseline factors for OS in univariate analyses are presented in (Supplementary Table 1). In univariate analysis HR for OS for right-sided primary tumour (others as reference) was 1.82 (95% CI 1.24–2.67) for *RAS* and *BRAF*wt, 1.25 (1.00–1.55) for *RAS*mt and 1.14 (0.70–1.85) for *BRAF*mt. In the multivariable analysis of prognostic factors for OS (Supplementary Table 1) metastasectomy and/or LAT was the strongest factor associated with survival (HR 0.24). The second most notable factor was the mutational status with *BRAF*mt (HR 2.39) and *RAS*mt (HR 1.54). Poor ECOG performance status, right-sided primary tumour and presence of baseline liver, peritoneal or suprarenal metastases were also associated with impaired OS.

## Discussion

Based on the repeated centralised assessment of resectability of multisite metastases [14, 15], this study demonstrated high upfront resectability (32% vs 29% vs 15%), conversion (23% vs 19% vs 8%) and resection/LAT (45% vs 37% vs 17%) rates in our real-world clinical material, in *RAS* and *BRAF*wt, *RAS*mt and *BRAF*mt patients, respectively. These figures were highly dependent on mutational status. Upfront resectability rates are rarely reported in the literature, see review in ref. [14], and to the best of our knowledge upfront resectability by mutational status has not previously been reported for non-selected mCRC patients.

In mCRC study populations *RAS*wt rates have been reported to be in the range of 40–54% [2, 18], slightly higher than the rate observed in this study, excluding patients who were not accurately *RAS*wt. In contrast, the *RAS*mt rates observed in our study were higher than those previously presented in the literature at 40–51% [2, 18]. In population-based series of unresectable patients, *BRAF*mt rates of 21% have been noted. We observed a *BRAF*mt rate of 10%, a number which is more in line with the rates observed in study populations of 5–10% [2, 3].

The SEER database shows that the liver is the most common site for CRC metastases (74%), followed by the lung (22%) [19], figures well in line with our data. However, no data according to molecular status is presented in that study [19]. *KRAS* mutations are associated with a higher prevalence and a more aggressive form of lung metastases [20], the former also supported by our data. Also, *BRAF*mt patients have more peritoneal and lymph node metastases but fewer liver and lung metastases than *BRAF*wt [3, 21], in line with our peritoneal metastasis findings. In a large Swedish national mCRC cohort, tumours with mucinous or signet cell histology have more peritoneal and fewer liver metastases, but the mutational status was not reported [22]. *BRAF*mt associates with this histology in our material, which could at least partially explain the Swedish findings.

Folprecht reported that response rates for combination chemotherapy correlated with conversion rates, and later verified “the higher the response, the better the conversion rate” for the addition of cetuximab to combination chemotherapy in *KRAS*wt disease [23, 24]. In the TRIBE-study of combination chemotherapy with bevacizumab, response rates and OS varied according to *RAS* and *BRAF* status, but conversion rates according to *RAS* and *BRAF* status were not reported [25]. Studies of triplet chemotherapy plus biologics for the treatment of borderline resectable patients have noted high response (81–87%) and conversion (33–61%) rates for liver-limited patients treated with triplet chemotherapy plus bevacizumab [26], or panitumumab [27]. In a study that used hepatic arterial infusion for unresectable CRC liver metastases. Datta et al. reported conversion rates of 45% in *RAS* and *BRAF*wt ± *TP53*mt, 45% in *RAS*mt + *TP53*wt, 39% in *RAS*mt + *TP53*mt, and no conversions for *BRAF*mt patients [28]. Further, a conversion rate of 22% for *RAS*wt was observed in the FIRE-3 trial [29]. In a Scandinavian population-based study conversion and resection rates of 11% in *KRAS* and *BRAF*wt, 8% in *KRAS*mt and only 1% in *BRAF*mt were reported [3]. Taken together, these results correspond well with our observation that the highest conversion rates were seen in *RAS* and *BRAF*wt, were almost as high in *RAS*mt and were clearly lower in *BRAF*mt, with the caveat of inclusion of multiple and multisite metastases in our real-world study.

The aforementioned publications that refer to resection rates for molecular subtypes mostly focus on initially unresectable or borderline diseases. We are not aware of other population-based series that report total resectability rates for all treatable mCRC patients according to *RAS* and *BRAF* status. Recently, a retrospective series of liver metastases noted a 34% resection/LAT rate in treated *BRAF*mt patients [30].

In addition to our RAXO group [14, 15], central resectability assessment for CRC liver metastases has been described by Huiskens [31], and for mCRC by Modest [32]. Both of these studies reported a high level of disagreement in evaluation, supporting the use of specialised MDT assessment without segregation for mutational status. When comparing central and local assessments of upfront resectability in our study, there were considerable discrepancies of 40–47% in *RAS* and *BRAF*wt and *RAS*mt, but as high as 69% in *BRAF*mt. *BRAF*mt patients in our study as in the literature [3], had poorer ECOG performance status and a metastatic profile more difficult for resection. Therefore, local pessimism is understandable, but undesirable as there were patients in this group who derived long-term benefits from resection. The repeated central assessment of technical resectability was performed without knowledge of mutational status, which probably partly explains this discrepancy in *BRAF*mt. The implications of a discrepancy between the central and local review of resectability status need to be addressed separately in each country. Resectability should repeatedly be addressed in organ-specific MDTs with significant experience in conversion treatments and challenging resections and/or LATs.

Outcome after resection and/or LAT in this study was excellent for *RAS* and *BRAF*wt (OS of 83 months) and very good for *RAS*mt (60 months), while it was modest for BRAFmt (30 months). In a review from Tsilimiras [33], 24 liver resection studies reported *RAS*mt as a negative prognostic factor for OS, in line with our study including all metastatic sites, whereas four studies found no effect of *RAS* status on OS. In these studies, OS was over 70 months for *RAS*wt and 20–51 months for RASmt, somewhat shorter than in our study. Twelve studies included in the review reported impaired outcomes for patients with *BRAF*mt, in line with our findings. Also, a meta-analysis of 11 prospective and retrospective studies of liver resection reported that *KRAS*mt and *BRAF*mt mutational status was negatively associated with OS and relapse-free survival (RFS) [9]. Also, a recent retrospective real-world study from US reported worse survival for *BRAF*mt mCRC patients compared to *BRAF*wt [34]. The worse outcome in *RAS*mt and *BRAF*mt is probably due to both the mutations and right-sided primary according to multivariable analysis. Sidedness affects metastatic profile, with less resectable metastatic sites in *BRAF*mt. In univariate analysis there was no significant OS difference for *BRAF*mt with right versus left-sided primaries. Despite the worse prognosis, long-term survival without relapse is still possible for *BRAF*mt after liver resection [13], and longer OS in *BRAF*mt is observed after resection than with systemic therapy only [30].

Patients with *RAS*mt or *BRAF*mt and synchronous CRC liver metastases have worse survival after resection than patients with wild-type tumours, but this difference is not observed in the case of metachronous metastases [35]. This may describe the more indolent nature of the metachronous disease and could be one factor favouring the decision to perform a resection. In a retrospective analysis of patients treated with any metastasectomy, *RAS*mt and the presence of liver metastases were the only independent risk factors of the impaired OS with a 4-year OS-rate of 81% for *RAS*wt versus 60% for *RAS*mt [36]. This is in line with our multivariate findings for *RAS* and *BRAF* status and liver metastases, but not for synchronous presentation.

Lung metastases themselves may not present the decisive factor for survival, and the role of pulmonary metastasectomy is not fully clear based on the PulMiCC study [37]. As a part of complete clearance of the disease, lung resection provides a possible cure in mCRC, as is also noted in the ESMO guidelines [5]. A recent meta-analysis in pulmonary resection reported impaired OS and RFS for *RAS*mt versus *RAS*wt patients, and similarly *BRAF*mt patients had worse survival than *BRAF*wt [20]. This is well in line with our findings and a favourable molecular profile could provide support for the decision of whether or not to perform lung resection.

Some studies have identified *RAS*mt as a negative prognostic marker after cytoreductive surgery with hyperthermic intraperitoneal chemotherapy (HIPEC) for peritoneal metastasectomy [38], but a recent Norwegian study found a similar OS of around 49 months after cytoreductive surgery with HIPEC irrespective of *RAS* and *BRAF* status [39]. In the latter study a *BRAF*mt subgroup with dMMR had superior survival among the patients with *BRAF*mt and this has also been reported in an unselected CRC cohort [40]. In line with these findings, we observed impaired survival for *BRAF*mt patients with peritoneal metastases, with the caveat that our study only included small patient numbers in this group. In addition, we had MMR analysis available from a fraction of the patients and therefore cannot compare these results.

The major strength of this study is the analysis of a complete set of data from 906 prospective real-world mCRC patients that were all considered treatable and, thus, the results are applicable to our everyday practice. Secondly, molecular pathology was mostly assessed as part of the clinical routine with accredited methods thus optimising systemic treatment choices regarding chemotherapy and biologics. Third, a repeated central assessment of resectability was performed at a tertiary centre maximising resectability, conversion and resection rate analyses. Fourth, central assessments were performed without knowledge of molecular status, making bias due to pre-knowledge of the potentially negative prognosis of *BRAF* and *RAS* mutants unlikely. Fifth, we included all metastatic sites in the resectability assessment and recorded sites and resections throughout the disease trajectory.

A clear limitation of this study is the observational design without any randomisation. Secondly, we had only 15 resected *BRAF*mt patients making confidence intervals wide. Given the often more widespread and aggressive nature of *BRAF*mt mCRC, this will be a problem in all prospective studies comparing different molecular subtypes. One way of overcoming this is conducting *BRAF*mt only studies such as the BEACON-study [41], although that specific study only concentrated on unresectable diseases. Large collaborative registries for *BRAF*mt would be of uttermost importance in overcoming this limitation. Third, MMR status was missing for 68% of patients, as testing was not recommended until the ESMO recommendations were updated in 2016 [5]. Fourth, all but the liver resection subgroups were quite small for robust comparisons. Fifth, we cannot currently provide accurate enough systemic treatment information per RAS/BRAF group. We are collecting later line treatment information and validating the correct use of biologics in the entire cohort of treatable patients.

In conclusion, there were significant differences in metastatic profile, resectability, conversion and resection/LAT rates according to *RAS* and *BRAF* status. Repeated centralised MDT assessment gives all patients an optimal chance for the best possible treatment. Outcomes for patients with multisite and multiple metastases were significantly better for *RAS* and *BRAF* wild-type compared with either mutant. Even *BRAF*mt patients have a chance of long-term survival with resection. Patients only receiving systemic therapy still have poorer long-term survival than resected patients, similarly, varying according to molecular status.

### Reporting summary

Further information on research design is available in the Nature Research Reporting Summary linked to this article.

## Supplementary information

Reporting Summary checklist

REMARK checklist

STROBE checklist

Supplementary information

## Data Availability

The data collected for this study can be made available to others in de-identified form after all primary and secondary endpoints have been published, in the presence of a data transfer agreement and if the purpose of use complies with Finnish legislation. Requests for data sharing can be made to the last author, including a proposal that must be approved by the steering committee.
