# Supplementary material for: Resectability, conversion, metastasectomy and outcome according to RAS and BRAF status for metastatic colorectal cancer in the prospective RAXO study
Source: Br J Cancer. 2022 May 24;127(4):686–94. doi: 10.1038/s41416-022-01858-8 (PMC9381729; doi:10.1038/s41416-022-01858-8)
Supplement: Supplementary file 4 — Supplementary information [file 41416_2022_1858_MOESM4_ESM.docx]

**Resectability, conversion, metastasectomy, and outcome according to *RAS* and *BRAF* status for metastatic colorectal cancer in the prospective RAXO study**

## Supplementary information

## The RAXO Study Group Investigators

*Helsinki University Hospital*

Pia Österlund, Helena Isoniemi, Aki Uutela, Leena-Maija Soveri, Päivi Halonen, Arno Nordin, Heikki Mäkisalo, Riikka Huuhtanen, Eila Lantto, Ali Ovissi, Juhani Kosunen, Sirpa Leppä, Petri Bono, Johanna Mattson, Jari Räsänen, Anna Lepistö, Emerik Österlund, Heidi Penttinen, Siru Mäkelä, Ari Ristimäki, Olli Carpén, Eila Lantto, Nina Lundbom, Antti Hakkarainen, Marjut Timonen.

*Tampere University Hospital*

Tapio Salminen, Pia Österlund, Kaisa Lehtomäki, Veera Salminen, Niina Paunu, Irina Rinta-Kiikka, Martine Vornanen, Nieminen Lasse.

*Turku University Hospital*

Annika Ålgars, Raija Ristamäki, Eetu Heervä, Johanna Virtanen, Eija Korkeila, Eija Sutinen, Maija Lavonius, Jari Sundström, Roberto Blanco

*Oulu University Hospital*

Raija Kallio, Markus Mäkinen, Eija Pääkkö

*Kuopio University Hospital*

Annamarja Lamminmäki, Hanna Stedt, Tiina Tuomisto-Huttunen, Päivi Auvinen, Vesa Kärjä, Sakari Kainulainen, Hannu-Pekka Kettunen

*Central Finland Central Hospital*

Ilmo Kellokumpu, Markku Aarnio, Ville Väyrynen, Kaija Vasala, Juha Kononen, Sanna Ketola, Teijo Kuopio, Kyösti Nuorva

*Satakunta Central Hospital*

Pia Österlund, Maija-Leena Murashev, Kalevi Pulkkanen, Venla Viitanen, Marko Nieppola, Elina Haalisto

*Päijät-Häme Central Hospital*

Paul Nyandoto, Aino Aalto

*South Pohjanmaa Central Hospital*

Timo Ala-Luhtala, Jukka Tuominiemi

*Kymenlaakso Central Hospital*

Anneli Sainast, Timo Muhonen, Laura Pusa, Sanna Kosonen, Leena Helle, Terhi Hermansson

*Kanta-Häme Central Hospital*

Riitta Kokko, Laura Aroviita, Petri Nokisalmi

*North Karelia Central Hospital*

Liisa Sailas, Heikki Tokola

*Vaasa Central Hospital*

Antti Jekunen, Teemu Pöytäkangas

*South Carelia Central Hospital*

Kari Möykkynen, Sanna Kosonen, Timo Muhonen

*Lapland Central Hospital*

Olli-Pekka Isokangas, Svea Vaarala

*South Savo Central Hospital*

Terhi Hermansson, Tuula Klaavuniemi, Rainer Kolle

*Kainuu Central Hospital*

Raija Kallio, Peeter Karihtala, Mirja Heikkinen

*Central Ostrobothnia Central Hospital*

Kaisu Johansson, Anna Sjöstrand, Piia Kajasviita

*Länsi-Pohja central hospital*

Jaana Kaleva-Kerola

*East Savo Central Hospital*

Esa Männistö

*Åland Central Hospital*

Reneé Lindvall-Andersson, Tom Kaunismaa, Pia Vihinen, Nina Cavalli-Björkman

## Supplementary Table 1. Multivariable analysis for risk factors of overall survival

|  |  | Univariate* | | Multivariable* | |
| --- | --- | --- | --- | --- | --- |
|  |  | Hazard ratio | CI^95%^ | Hazard ratio | CI^95%^ |
| Age > 70 years |  | 1.22 | 1.03-1.44 | 1.01 | 0.43-1.21 |
| Female sex |  | 1.03 | 0.87-1.21 |  |  |
| ECOG score | PS 0 | ref. |  | ref. |  |
|  | PS 1 | 1.73 | 1.42-2.11 | 1.46 | 1.19-1.79 |
|  | PS 2-3 | 3.27 | 2.56-4.19 | 2.23 | 1.72-2.89 |
| Charlson comorbidity index | 0 | ref. |  | ref. |  |
|  | 1-2 | 1.22 | 1.01-1.48 | 1.04 | 0.86-1.27 |
|  | 3-5 | 1.46 | 0.60-3.52 | 1.38 | 0.57-3.38 |
| Body mass index | <20 | ref. |  |  |  |
|  | 20-30 | 1.03 | 0.75-1.41 |  |  |
|  | >30 | 0.84 | 0.59-1.20 |  |  |
| Primary tumour in right colon |  | 1.60 | 1.35-1.89 | 1.25 | 1.03-1.51 |
| Synchronous metastases§ |  | 1.47 | 1.23-1.75 | 1.05 | 0.86-1.29 |
| Metastatic organs at baseline | Liver metastases | 1.24 | 1.03-1.49 | 1.86 | 1.45-2.39 |
|  | Lung metastases | 1.36 | 1.15-1.61 | 0.94 | 0.75-1.17 |
|  | Distant lymph node metastases | 1.74 | 1.49-2.06 | 1.18 | 0.93-1.48 |
|  | Peritoneal metastases | 1.61 | 1.32-1.96 | 1.40 | 1.08-1.83 |
|  | Local relapse | 1.18 | 0.86-1.61 |  |  |
|  | Bone metastases | 3.33 | 2.10-5.28 | 1.40 | 0.85-2.31 |
|  | Ovarian metastases | 1.42 | 0.90-2.43 |  |  |
|  | Suprarenal metastases | 3.16 | 1.78-5.61 | 2.23 | 1.21-4.10 |
|  | Brain metastases | 1.87 | 0.47-7.50 |  |  |
| More than one metastatic site at diagnosis of mCRC |  | 2.07 | 1.76-2.43 | 1.12 | 0.87-1.45 |
| Mutational status | *RAS*&*BRAF* wild type | ref. |  | ref. |  |
|  | *RAS* mutation | 1.49 | 1.24-1.79 | 1.54 | 1.27-1.87 |
|  | *BRAF* mutation | 3.19 | 2.42-4.21 | 2.39 | 1.76-3.25 |
| Metastases treated with resection or LAT |  | 0.19 | 0.15-0.23 | 0.22 | 0.17-0.27 |
| * Variables significant in univariate analysis entered in multivariable analysis.  § Within 2 months from the diagnosis of primary tumour.  Ref. Reference category.  mCRC = metastatic colorectal cancer.  LAT = local ablative therapy as radiofrequency ablation or stereotactic radiotherapy. | | | | | |


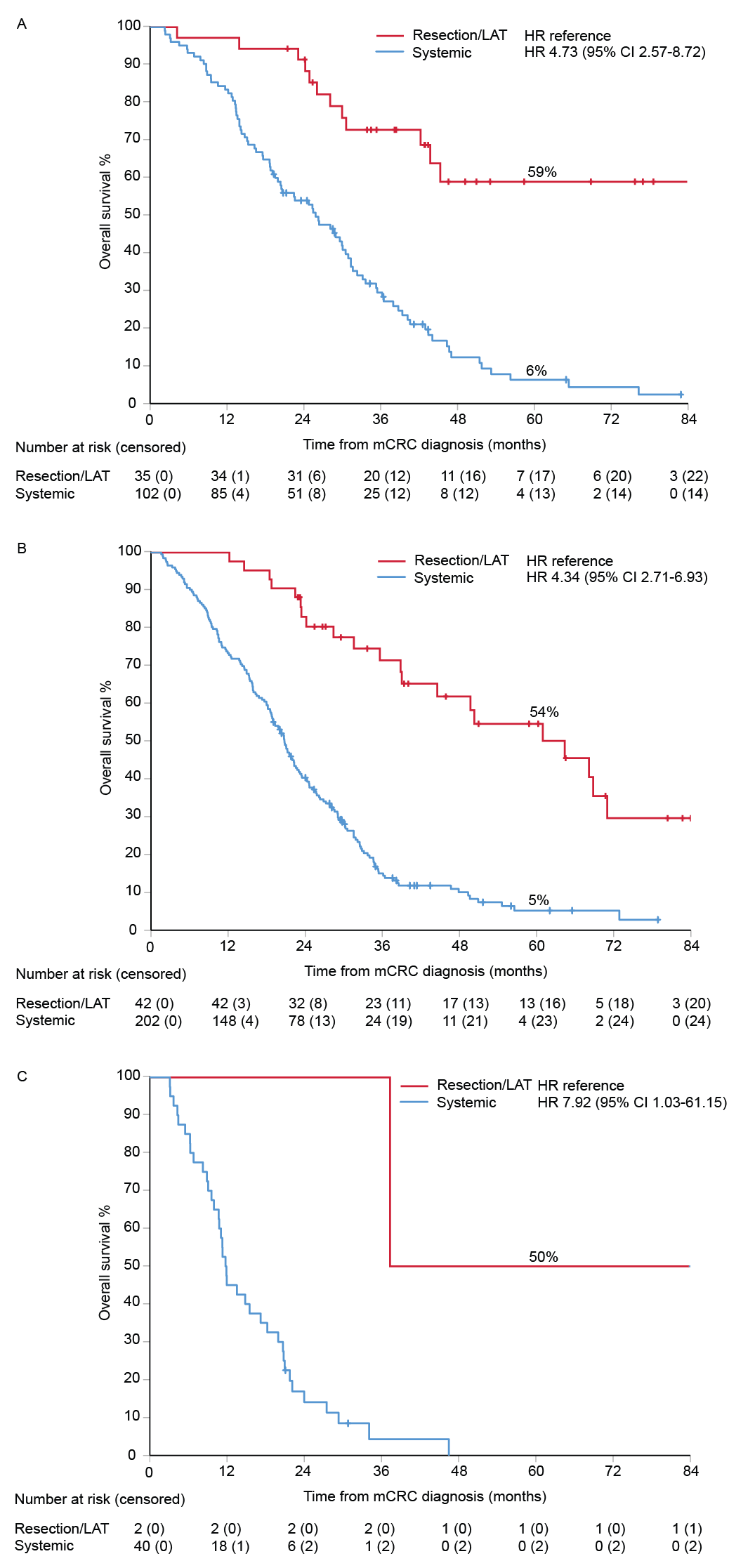


## Supplementary Figure 1. Survival of patients with multiple metastatic sites according to mutational and resection status. A. *RAS*&*BRAF* wild type (wt). B. *RAS* mutated type (mt). C. *BRAF*mt


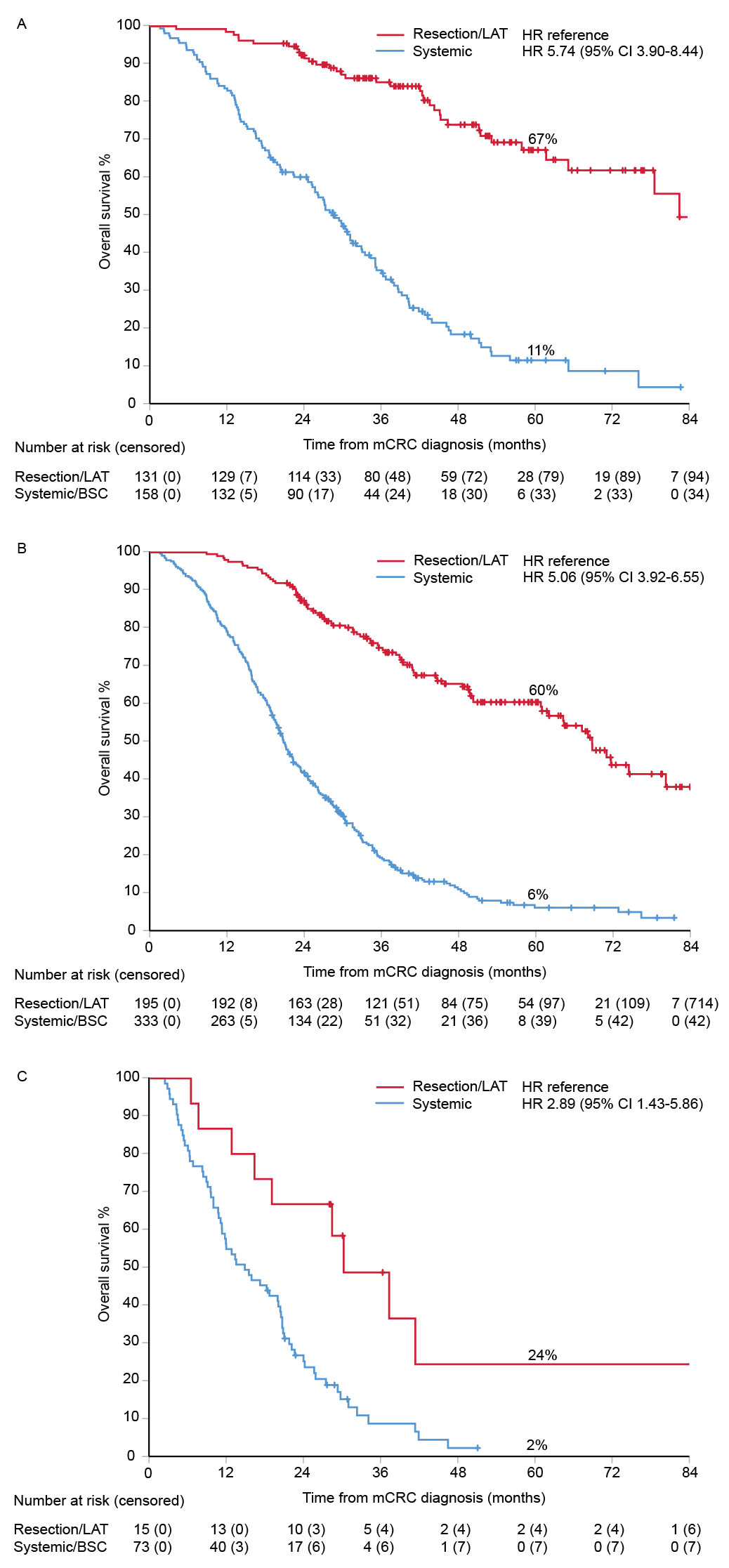


## Supplementary Figure 2. Overall survival for patients resected or not resected according to mutational status A. *RAS*&*BRAF*wt. B. *RAS*mt. C. *BRAF*mt


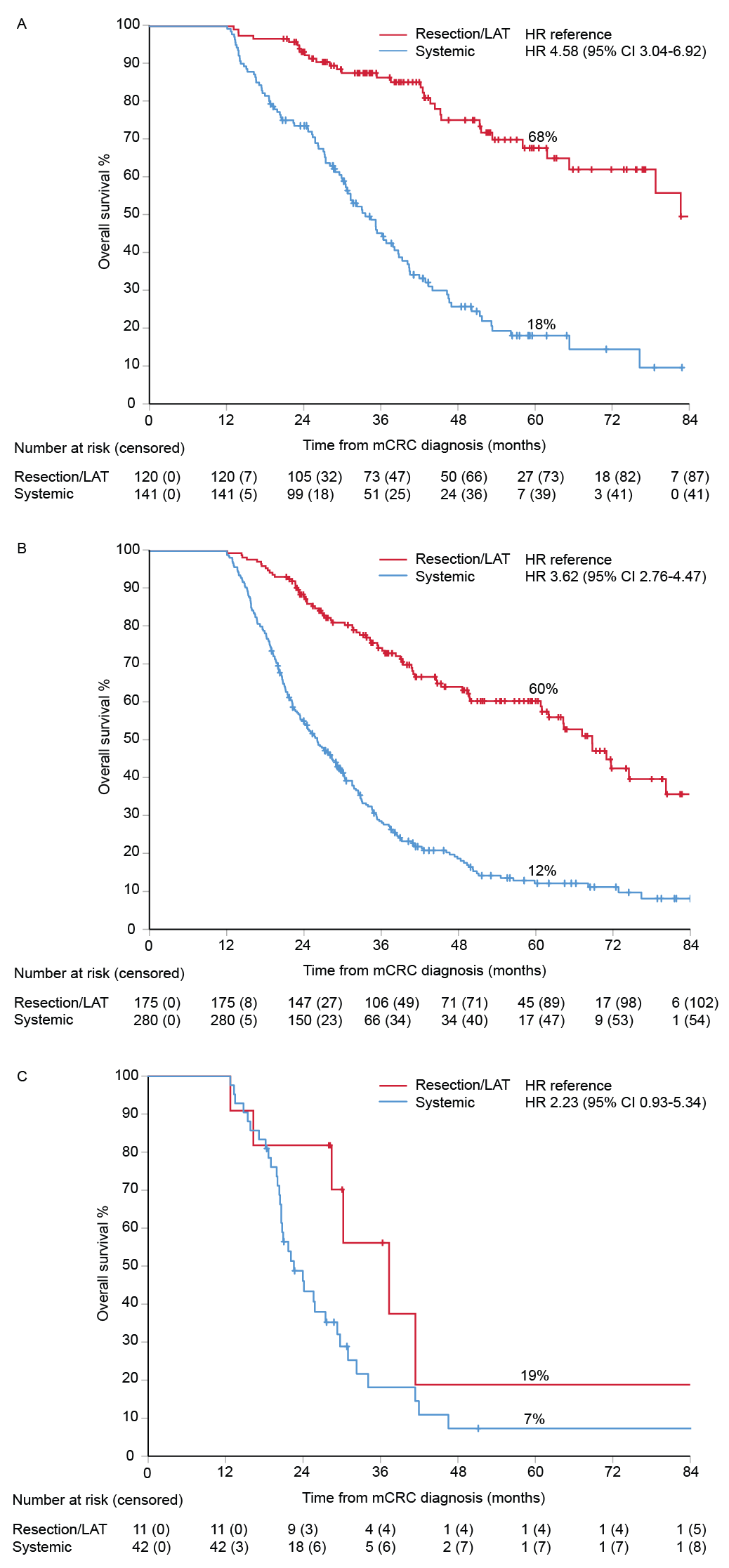


## Supplementary Figure 3. 12-month conditional Landmark analysis of overall survival. A. *RAS*&*BRAF*wt. B. *RAS*mt. C. *BRAF*mt
